# Reassessing Agreement Between Point of Care and Laboratory Triglyceride Measurements in High Risk Pregnancy

**DOI:** 10.1002/edm2.70272

**Published:** 2026-07-14

**Authors:** Faizan Ahmed, Ahmed Raza, Abdullah Aman, Shahzadi Gulfishan

**Affiliations:** ^1^ Shaikh Khalifa Bin Zayed Al‐Nahyan Medical College Lahore Pakistan; ^2^ Pakistan Institute of Medical Sciences Islamabad Pakistan; ^3^ Riphah International University Islamabad Pakistan

Dear Editor,

We read with great interest the article by Farabi et al. entitled Portable Home‐Use Triglyceride Meter Demonstrates Good Agreement with Plasma Triglycerides in Pregnant Women with Obesity and Gestational Diabetes Mellitus [1]. The study addresses an important and clinically relevant challenge, as maternal hypertriglyceridemia has increasingly been implicated in adverse pregnancy outcomes, including fetal overgrowth and excess neonatal adiposity. The authors should be commended for evaluating a practical point‐of‐care approach that may facilitate more accessible triglyceride monitoring during pregnancy. However, several methodological considerations warrant further discussion, as they may influence the interpretation of the reported agreement between capillary and laboratory triglyceride measurements.

First, although 68 fasting samples were analysed, these measurements were obtained from only 35 participants, with several women contributing repeated observations. The statistical analyses, including intraclass correlation coefficients (ICCs) and Bland–Altman plots, appear to have treated these observations as independent. Failure to account for within‐subject correlation may underestimate variability and potentially inflate measures of agreement, thereby overestimating the reliability of the POC device. Future studies should employ mixed‐effects models or repeated‐measures agreement analyses specifically designed for replicated observations [2].

Second, women with triglyceride concentrations ≥ 400 mg/dL were excluded from the analysis. While this criterion may have been necessary for methodological reasons, it limits evaluation of device performance across the full spectrum of clinically relevant triglyceride values. Agreement between measurement methods may vary at higher analyte concentrations, particularly among individuals at greatest metabolic risk. Inclusion of participants with severe hypertriglyceridemia and stratified analyses across triglyceride ranges would enhance the generalisability of the findings and provide a more comprehensive assessment of device accuracy [3].

Third, the authors propose the device as a potential home‐monitoring tool; however, all measurements were obtained by trained research personnel under controlled study conditions. Consequently, the reported agreement may not reflect real‐world performance during unsupervised patient use, where factors such as sampling technique, device handling, and user‐related variability may influence accuracy. Future investigations should evaluate device performance in home settings and incorporate usability assessments to determine whether the observed agreement is maintained in routine practice [4].

Finally, although the reported ICC values suggest a strong association between methods, correlation‐based measures alone do not establish clinical interchangeability. Notably, the Bland–Altman analyses demonstrated relatively wide limits of agreement, indicating the possibility of substantial differences between methods at the individual‐patient level. Without predefined clinical acceptability thresholds, it remains uncertain whether these differences are sufficiently small to support interchangeable clinical use. Future studies should incorporate clinically meaningful agreement criteria and evaluate concordance using established method‐comparison frameworks [5, 6].

In Conclusion, despite these considerations, the study represents an important step toward expanding access to lipid monitoring during pregnancy. Addressing these methodological issues in future research may further strengthen the evidence supporting the clinical implementation of POC triglyceride testing.

## Author Contributions

**Abdullah Aman:** resources, writing – original draft. **Faizan Ahmed:** writing – original draft. **Shahzadi Gulfishan:** resources, writing – review and editing. **Ahmed Raza:** supervision, writing – review and editing.

## Funding

The authors have nothing to report.

## Disclosure

Guarantor Statement: All authors have read and approved the final version of the manuscript. They took complete responsibility for the data's integrity and the accuracy of the data analysis.

Transparency Statement: The Authors affirm that this manuscript is an honest, accurate, and transparent account of the study being reported, that no important aspects of the study have been omitted, and that any discrepancies from the study as planned (and if relevant, registered) have been explained.

## Ethics Statement

The authors have nothing to report.

## Conflicts of Interest

The authors declare no conflicts of interest.

## Data Availability

Data sharing does not apply to this article as no datasets were generated during the current study; all data were sourced from published literature.
